# Effects of dietary supplementation with breadfruit leaf powder on growth performance, meat quality, and antioxidative activity in Japanese quail

**DOI:** 10.14202/vetworld.2021.1946-1953

**Published:** 2021-07-28

**Authors:** Muhammad Thohawi Elziyad Purnama, Eric Putra Ernanda, Faisal Fikri, Agus Purnomo, Shafia Khairani, Shekhar Chhetri

**Affiliations:** 1 Division of Veterinary Anatomy, Department of Veterinary Science, Faculty of Veterinary Medicine, Universitas Airlangga, Surabaya, Indonesia; 2 Division of Veterinary Clinical Pathology and Physiology, Department of Veterinary Science, Faculty of Veterinary Medicine, Universitas Airlangga, Surabaya, Indonesia; 3 Department of Veterinary Surgery and Radiology, Faculty of Veterinary Medicine, Universitas Gadjah Mada, Yogyakarta, Indonesia; 4 Department of Biomedical Science, Faculty of Medicine, Universitas Padjajaran, Bandung, Indonesia; 5 Department of Animal Science, College of Natural Resources, Royal University of Bhutan, Lobesa, Punakha, Bhutan

**Keywords:** antioxidative activity, breadfruit leaf powder, food availability, growth performance, Japanese quail, meat quality

## Abstract

**Background and Aim:**

In an era of increasing concerns about food availability globally, poultry meat is being increasingly consumed rather than red meat given its quality in terms of pH, color, and tenderness, conferring consumer satisfaction. The choice of feed is a crucial factor in poultry production. This study investigated the effect of dietary supplementation with breadfruit leaf powder on growth performance, meat quality, and antioxidative activity in Japanese quail.

**Materials and Methods:**

A total of 120 day-old quail were used in this study and assigned equally into four treatment groups: Group C fed a basal diet and three treatment groups fed a basal diet supplemented with 2.5% (T1), 5% (T2), or 10% (T3) breadfruit leaf powder. The concentrations of breadfruit leaf powder were 2.5, 5, and 10 g/kg in the basal diet. Quail body weight and feed intake (FI) were evaluated at 1, 21, and 35 days of age at 7 a.m. Pectoral muscle was collected to determine pH, meat color, drip loss, cooking loss, water-holding capacity (WHC), tenderness, and antioxidant levels. All variables were analyzed statistically using ANOVA followed by Duncan’s *post hoc* test (significance set at p<0.05).

**Results:**

T3 showed increased body weight gain of quails at1-21 and 21-35 days (p<0.05). Feeding in the T3 group improved the feed conversion ratio compared with those in the C and T1 groups at the starter phase (p<0.05). Dietary treatment did not affect FI (p>0.05). In the present study, meat redness and WHC were improved in the T3 group (p<0.05). Meanwhile, drip loss, cooking loss, and meat tenderness were improved in the T2 group (p<0.05). The pH_45 min_, pH_24 h_, lightness, and yellowness were not influenced by the treatments (p>0.05). The antioxidative activities of superoxide dismutase and malondialdehyde decreased in the T3 group (p<0.05), while no significant difference in glutathione peroxidase level (p>0.05) was identified.

**Conclusion:**

Ten grams/kilogram of breadfruit leaf powder, as administered in the T3 group, can be applied as a dietary supplement for Japanese quail to improve growth performance, meat quality, and antioxidative activity during the starter and grower periods.

## Introduction

In recent years, the consumption of poultry meat in Indonesia has increased. The popularity of poultry on the market has also shown a tendency to increase compared with that of beef [1]. Japanese quail (*Coturnix coturnix japonica*) is a poultry commodity that is popular with consumers. Quail is used for two purposes: To produce eggs and meat [2]. Several studies have been conducted to determine the health benefits of quail meat and its meat quality compared with those of other poultry [3]. Quail rapidly reaches sexual maturity, resistance to environmental temperature and starting to adapt to maintaining body weight t 3-5 weeks of age. Male quail can be harvested at 5-6 weeks of age with a body weight of 100-140 g and acarcass percentage of 73.33% [4]. Moreover, in production, adult quail can be harvested at 6-7 weeks with a weight of 300 g and a carcass range of 75-78% [5].

Consumer satisfaction is often achieved if meat has a soft texture [6]. The main components of meat that play an important role in the level of tenderness are connective tissue, muscle fibers, and fat tissue [7]. Less connective tissue in meat confers a soft texture on it. With higher levels of marbling fat or intramuscular fat, the texture of meat becomes more tender. The presence of intramuscular fat in muscle loosens the muscle fiber bonds, thus providing opportunities for meat protein to bind water [8].

Factors that can affect the water-binding capacity (water-holding capacity [WHC]) are pH, muscle type and location, muscle function, age, feed, rigor mortis phase, and intramuscular fat level and species [9]. The WHC increases in direct proportion to the pH of meat. A low pH of meat can decrease its WHC [10]. pH levels, length of muscle sarcomeres, pieces of muscle fibers, myofibril contraction, and size and weight of meat samples affect the cooking losses, which are in the range of about 1.5-54.5%. Quail meat has a dark color since the ratio of dark to light muscles in quail breast meat is 95.1-96.7%:3.7-4.9%. This predominance of dark meat in quail confers a tougher texture, as dark meat is tougher than light meat [11]. There is thus a need to formulate an additional feed to tenderize quail meat.

Breadfruit *(Artocarpus altilis*) is a versatile plant because all parts of itcan be used for medicinal plants. The leaves of breadfruit contain various natural antioxidants, such as flavonoids, carotenoids, hydrocyanic acid, acetylcholine, tannins, riboflavins, saponins, phenols, quercetin, kaempferol, potassium, and Vitamins A, E, and C [12]. The antioxidant status of feed is associated with meat quality [13]. The aforementioned components of breadfruit leaves are expected to improve meat quality and tenderness through antioxidant activity in quail meat. The previous studies on breadfruit leaves proved that they can be used as a fattening formula [14].

This study aimed to evaluate the effects of dietary supplementation with breadfruit leaf powder on the growth performance, meat quality, and antioxidative activity in Japanese quail.

## Materials and Methods

### Ethical approval

This study was approved by Ethical Committee of Animal Care and Use No.768/HRECC.FODM/XII/2019 Universitas Airlangga. Experimental animals were healthy male sexed day-old quail (DOQ) purchased from private breeding farm.

### Study period and location

This study was conducted for 2 months (September and October 2019). The DOQ was reared in a private breeding farm. Proximate analysis was evaluated at laboratory animal nutrition, Faculty of Veterinary Medicine, Universitas Airlangga. Meat examinations were performed at the Laboratory of Animal Production, PSDKU Banyuwangi, Universitas Airlangga. The antioxidative parameter was evaluated at Gamma Scientific Biolab, Malang, East Java.

### Experimental design

A total of 120 DOQ were reared and equally assigned into four treatment groups. The control (C) group was fed with a basal diet, while the treatment groups were fed a basal diet supplemented with 2.5% (T1), 5% (T2), or 10% breadfruit leaf powder (T3). The concentrations of breadfruit leaf powder were 2.5, 5, and 10 g/kg in the basal diet. The basal diet components and nutrient contents are shown in Table-1. The treatments were applied in the starter phase (1-21 days) and grower phase (21-35 days).

**Table-1:** Feed ingredients and nutrients content of basal diets.

Ingredients (g/kg)	Diets		Nutrients	Diets	
	
	Starter phase (1-21 days)	Grower phase (21-35 days)		Starter phase (1-21 days)	Grower phase (21-35 days)
Corn	530	589	Metabolite energy (MJ/kg)	12.98	12.98
Soybean meal	336	322	Crude protein (g/kg)	227	206
Corn oil	60	50	Calcium (g/kg)	10	9.1
Dicalcium phosphate	16	16	Phosphorus (g/kg)	7.1	6.6
Calcium carbonate	17	13	Methionine and cystine	9.0	6.3
Methionine	2.0	1.0	Lysine (g/kg)	11.8	10
Vitamin premix	25	25			
a. Vitamin A (IU)	15000	15000			
b. Vitamin D3 (IU)	3750	3750			
c. Vitamin E (mg)	37.5	37.5			
d. Vitamin K3 (mg)	2.55	2.55			
e. Thiamin (mg)	3	3			
f. Riboflavin (mg)	7.5	7.5			
g. Vitamin B6 (mg)	4.5	4.5			
h. Vitamin B12 (µg)	24	24			
i. Niacin (mg)	51	51			
j. Folic acid (mg)	1.5	1.5			
k. Biotin (mg)	0.2	0.2			
l. Pantothenic acid (mg)	13.5	13.5			
m. Choline chloride (mg)	250	250			
n. Antioxidant (mg)	100	100			
Mineral mix	25	25			
a. Zinc (mg)	37.5	37.5			
b. Manganese (mg)	37.5	37.5			
c. Iron (mg)	37.5	37.5			
d. Copper (mg)	3.75	3.75			
e. Iodine (mg)	0.83	0.83			
f. Sulfur	62.5	62.5			
g. Selenium (mg)	0.23	0.23			
Salt	4.0	4.0			

Quail body weight and feed intake (FI) were evaluated per replicate on 1, 21, and 35 days of age at 7 a.m. The growth performance represented as FI, body weight gain (BWG), and feed conversion ratio (FCR) was evaluated in the starter phase (1-21 days), grower phase (21-35 days), and the whole period (1-35 days). Quails were not vaccinated during treatment. At the end of the study, quails were slaughtered and the pectoral muscle was collected in plastic bags for evaluation.

### Proximate analysis of breadfruit leaf powder

The breadfruit leaves (Herb No.074/338A) used in this study were identified from Glagah, Banyuwangi (8°10’31.1”S 114°17’07.5”E) by Mr. Husin, a botanist. The leaves were collected and then chopped, dried, and mashed into powder. The proximate analysis was performed in accordance with the AOAC method [15]. The carbohydrate content was determined by the formula [carbohydrate = feed mass - (ash + crude protein + fat + fiber)]. The ash content was evaluated using a muffle electric furnace by ashing the samples overnight at 600°C. The crude protein analysis was performed using the Kjeldahl method. The total fat analysis was performed using the soxhletation method. The results of proximate analysis of the experimental diets are shown in Table-2.

**Table-2:** Proximate analysis of the experimental diets.

Treatment	Contents (%)							

	Dry matter	Ash	Crude protein	Fat	Crude fiber	Calcium	Nitrogen-free extract material	Metabolic energy (kcal/kg)
C	90.03	5.77	20.27	5.25	4.21	2.23	54.52	3069.25
T1	89.89	6.25	20.20	5.66	4.97	3.45	52.80	3035.18
T2	89.92	6.60	18.99	5.95	6.91	3.49	51.48	2971.33
T3	89.94	7.16	18.33	7.84	7.02	3.66	49.59	3016.14

### Meat quality determination

The initial pH (at 45 min) and the final pH (24 h) after slaughter were recorded using a digital pH meter (EcoTestr, USA) with an accuracy of ±0.1 [16]. Meat color was determined using a chromatometer color reader (Konica Minolta, Japan) for the variables of L* (lightness), a* (redness), and b* (yellowness), according to the Commission Internationale de L’Eclairage (CIE) lab color system, on the posterior surface of the pectoral muscle. Color was measuredat three different sites [17].

Drip loss was analyzed by determining the initial weight (x) of the meat sample, which was then wrapped in a plastic bag and stored in a refrigerator for 24 h. The sample was weighed again to determine the final weight (y) by wiping with a tissue without pressing [18]. Drip loss was calculated using the following equation:


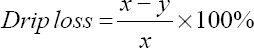


Frozen meat samples from the freezer were thawed at 4°Cfor 24 h and the initial weight (x) was recorded. The meat sample was cooked in a water bath at 85°C for 10 min [19]. Subsequently, the weight (y) of the meat samples was again recorded to calculate the cooking loss using the following equation:


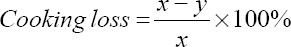


WHC was analyzed by homogenizing a 2 g sample of pectoral muscle with 4 mL of 0.6 M NaCl (x) in a tube. The homogenized samples were vortexed for 30 s and then incubated for 30 min at 4°C. Thereafter, each sample was centrifuged (Hettich EBA 200, Germany) at 2889 rpm for 30 min at 4°C [20]. The supernatant (y) was removed and WHC was calculated using the following equation:


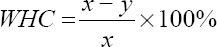


Tenderness was evaluated using a penetrometer. First, the sample was prepared by cutting the pectoral muscle to a size of 5´3´2 cm. The penetrometer needle was calibrated to the meat surface and it was inserted into 10 different sites. The result of this was indicated by a number on the penetrometer scale. The maximum pressing duration required was 10 s [21].

### Antioxidant activity evaluation

The samples were homogenized with a PRO 200 homogenizer (Pro Science, USA). Each homogenized sample was centrifuged at 10,000 rpm for 20 min at 4°C to obtain supernatants followed by storage at –20°C for subsequent analysis. The antioxidative activities of superoxide dismutase (SOD), malondialdehyde (MDA), and glutathione peroxidase (GPx) were determined using assay kits (Jiancheng Bioengineering Institute, China) [22].

### Statistical analysis

All data were expressed as mean±standard error and analyzed using one-way analysis of variance followed by *post hoc* Duncan’s comparison test. Values were considered significantly different at p<0.05. Statistical analysis was performed using SPSS v.25 software (IBM, USA).

## Results

### Growth performance

Growth performance was observed using the variables FI, BWG, and FCR during the starter phase, grower phase, and the whole period. In general, FI was not influenced by the experimental diet. Supplementation of 10 g/kg dietary breadfruit leaf powder (T3) caused an increase in BWG compared with that in the other treatment groups in all periods of treatment. Meanwhile, BWG in the T1 group showed no significant difference compared with that in the T2 and T3 groups at the grower phase (Table-3).

**Table-3:** Effects of experimental diets on growth performance in the end of treatment.

Variables	Treatment			

	C	T1	T2	T3
Feed intake (FI) (g)				
Starter phase	11.62±0.55	10.72±0.54	10.17±0.69	10.79±0.35
Grower phase	9.44±0.39	8.29±0.99	9.24±0.85	10.65±0.49
Whole period	10.97±0.46	9.99±0.65	9.89±0.62	10.75±0.32
Body weight gain (BWG) (g)				
Starter phase	1.83±0.02^b^	1.75±0.01^b^	1.78±0.02^b^	2.10±0.07^a^
Grower phase	2.62±0.16^b^	2.77±0.02^ab^	2.78±0.02^ab^	2.97±0.17^a^
Whole period	2.22±0.08^b^	2.26±0.01^b^	2.28±0.02^b^	2.54±0.11^a^
Feed conversion ratio (FCR)				
Starter phase	6.35±0.25^b^	6.12±0.29^b^	5.71±0.39^ab^	5.15±0.16^a^
Grower phase	3.72±0.29	2.99±0.36	3.31±0.31	3.72±0.35
Whole period	5.00±0.33	4.42±0.29	4.34±0.27	4.30±0.24
Initial weight (g)	7.53±0.13	7.55±0.11	7.63±0.09	7.61±0.08
Final weight (g)	125.76±3.81	120.33±2.83	116.19±2.99	122.56±4.28

Values are expressed in mean±standard error (n=30 animals for each four treatment groups). One-way analysis of variance was carried out followed by Duncan’s comparison test. ^a,b^Different superscripts in the same row indicate significant differences (p<0.05).

In addition, for FCR, the T3 group showed a significant difference compared with the C and T1 groups at the starter phase. From the grower phase and the whole period, no difference in FCR was observed. These results showed that supplementation of 10 g/kg dietary breadfruit leaf powder improved BWG and FCR at the starter phase. In particular, this concentration increased BWG at the grower phase and the whole period (Table-3).

### Meat quality

Meat quality was characterized using the following variables: pH_45 min_, pH_24 h_, meat color (lightness, redness, and yellowness), drip loss, cooking loss, WHC, and tenderness. When compared with the C group, there were no significant differences in pH_45 min_, pH_24 h_, lightness, and yellowness in all treatment groups. The T3 group presented significant differences compared with the other groups in terms of redness, drip loss, cooking loss, WHC, and tenderness. Meanwhile, the T2 and T3 groups showed similar observations regarding drip loss, cooking loss, and meat tenderness (Table-4). Based on the evaluation of meat quality traits, supplementation with 10 g/kg dietary breadfruit leaf powder improved the quality of quail meat during the treatment period.

**Table-4:** Effects of experimental diets on meat quality in quail.

Variables	Treatment			

	C	T1	T2	T3
pH_45 min_	6.19±0.03	6.17±0.02	6.19±0.02	6.19±0.02
pH_24 h_	6.13±0.03	6.08±0.02	6.11±0.02	6.10±0.02
Lightness	41.86±0.09	42.03±0.13	42.06±0.12	42.11±0.21
Redness	8.08±0.02^b^	8.14±0.14^b^	8.13±0.01^b^	8.46±0.02^a^
Yellowness	12.36±0.12	12.36±0.09	12.47±0.09	12.43±0.09
Drip loss (%)	21.54±0.09^c^	20.36±0.09^b^	19.59±0.16^a^	19.39±0.08^a^
Cooking loss (%)	22.65±0.07^c^	21.71±0.13^b^	20.64±0.16^a^	20.37±0.09^a^
WHC (%)	66.31±0.21^c^	66.44±0.24^c^	67.26±0.15^b^	68.41±0.19^a^
Tenderness (mm/g/10 s)	66.00±0.21^b^	67.20±0.29^b^	74.40±0.26^a^	74.20±0.28^a^

Values are expressed in mean±standard error (n=30 meat samples for each four treatment groups). One-way analysis of variance was carried out followed by Duncan’s comparison test. ^a,b,c^Different superscripts in the same row indicate significant differences (p<0.05).

### Antioxidant activity

**Figure-1:**
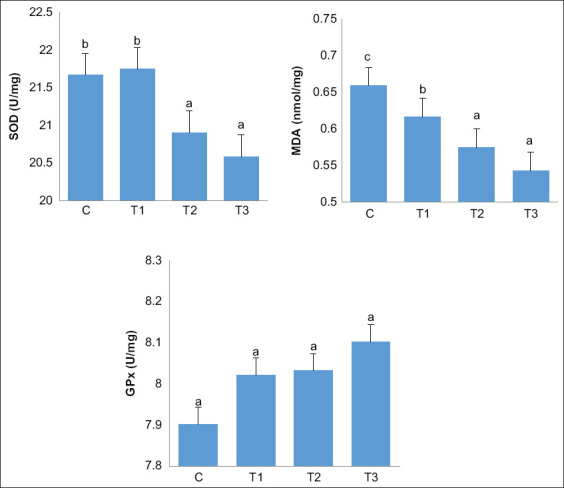
Effects of experimental diets on the antioxidative activities in quail meat. Values are expressed in mean±standard error (n=30 meat samples for each four treatment groups). One-way analysis of variance was carried out followed by Duncan’s comparison test. ^a,b,c^Different superscripts indicate significant differences (p<0.05). SOD=Superoxide dismutase, MDA=Malondialdehyde, GPx=Glutathione peroxidase.

In comparison to the findings in the other groups, the T3 group did not show a difference in GPx activity, but showed significant decreases in SOD and MDA activity. The results also showed a gradual improvement of SOD and MDA activity with progression of the experimental diet from T1 to T3 (Figure-1).

## Discussion

This study showed the efficacy of breadfruit leaf powder for increasing BWG and FCR, although no significant difference in FI was identified. Breadfruit leaf powder does not have a special taste to increase appetite, which is consistent with the previous studies showing that feeding on a breadfruit leaf powder diet did not increase the palatability of chickens [23]. The fat and crude protein components are proposed to increase the active protoplasm in muscle cells [24]. The proximate analysis in this study revealed fat and crude protein contents of 7.84% and 18.33%, respectively. A protein level of 16-18% is ideal to stimulate BWG during the grower phase [25]. Excess protein actually triggers a nitrogen imbalance, which results in high ammonia production [26]. Higher protein diets produce an increase in heat, which occurs due to N degradation resulting in the catabolism of amino acids into uric acid [27]. The limited capacity of the intestine to absorb amino acids can increase the excretion of amino acids in the urine [28].

The levels of calcium and metabolic energy available in feed are believed to accelerate growth and be efficient at improving FCR [29]. Calcium levels can maintain the development of muscle mass and stabilize actin and myosin activity during muscle contraction [30]. A lack of calcium in feed can increase the likelihood of hypocalcemia and paralysis in the extremities [31]. To maintain the growth phase, there is a need for metabolic energy in standard feed of at least 2880 kcal/kg [32]. Regarding the experimental diet in this study, it was shown to have a metabolic energy content consistent with minimum standards of 2880 kcal/kg. Metabolic energy can increase the FCR value and is positively correlated with BWG [33]. An increase in the FCR value was also revealed at the starter phase in this study.

In terms of meat quality, this study also reported on variables such as pH, color, freshness, and meat tenderness, which are crucial criteria for consumer satisfaction [34]. The pH of the meat of quail fed with breadfruit leaf powder showed no significant difference from that of the control group. Meat with pH of <5.7 is categorized as pale, soft, and exudative (PSE). Meanwhile, meat with a pH of >6.1 is categorized as dark, firm, and dry (DFD) [35]. The pH is also influenced by muscle glycogen catabolism by anaerobic glycolysis enzymes and lactic acid accumulation [36]. High lactic acid in meat results in a decrease in the postmortem pH. In addition, increased levels of lactic acid are supported by postmortem glycolysis activity by pyruvate kinase and lactate dehydrogenase [37,38].

Meat color is one of the most important quality criteria related to pH, WHC, and meat shear force [39]. Meat with a lightness value of >53 is categorized as PSE meat. The lightness of meat is considered to be normal when the score is 48-53. On the other hand, meat with a score of <46 is categorized as DFD meat [40]. PSE meat is influenced by protein denaturation in muscle and postmortem pH [37]. The present study reported that supplementation with breadfruit leaf powder had a significant effect on the redness of quail meat. Lightness and redness values are more idiosyncratic reflecting than yellowness values in poultry meat [41]. The redness value of meat depends on the redox reactions of myoglobin, hemoglobin, and heme pigment [42]. Meanwhile, yellowness in poultry is influenced by factors such as genetics, carotenoid pigments in feed, liver biochemistry, and meat processing [43].

In this study, the treatment groups differed significantly from the control group in terms of the variables of drip loss, cooking loss, WHC, and tenderness. Drip loss has the potential to occur at 24 h postmortem [44]. In general, drip loss is a process that involves the transfer of water from myofibrils in muscle tissue to extracellular tissue [45]. The level of drip loss is related to the functions of actin and myosin in the muscle after slaughter [46]. Quail that consumed the experimental diet had protoplasm reserves in muscle cells, which reduced protein denaturation. The availability of protoplasm increases the ability to bind water and prevents the release of water from meat [47]. Alleviation of the denaturation of myofibrillar and sarcoplasmic proteins can maintain muscle physiology [48], increase the ability of proteins to bind water, produce optimal WHC [49], and prevent the loss of soluble sarcoplasmic constituents from muscle cells to extracellular tissue [50]. The optimal percentage of WHC is supported by membrane integrity due to improvement in the level of creatine kinase and an increase in the hematocrit [51]. Here, there was a finding consistent with the previous studies that creatine kinase activity decreased with decreasing percentage of drip loss in broiler chickens [52].

Meat quality is also associated with oxidant and feed scavenger properties in different ways [53]. The antioxidant properties of breadfruit leaf powder are conferred through components such as phenolics and flavonoids [54], which react with lipid and hydroxyl radicals, and then convert them into resistant compounds [55]. Flavonoids can activate lipase, which plays a role in converting intramuscular fat into fatty acids and glycerol [56]. At lipolysis mechanism, which is initiated by lipase activity, this will then be followed by an increase in meat tenderness. Meat tenderness also depends on collagens. Meat tenderness has been reported to increase with decreasing intramuscular fat and collagen levels [57]. In addition, meat tenderness was shown to be related to myofibril protein, myofibril integrity, and rigor status [58].

In this study, regarding the antioxidant activity, there were improvements in SOD and MDA levels in the T3 group. On the other hand, GPx levels showed similar results in all treatment groups. Phenolic compounds inhibit lipid peroxidation, particularly unsaturated fatty acids [59], which leads to the antioxidant properties of the experimental feed [60]. Dietary supplements have been reported to modulate endogenous antioxidants and cell barriers [61]. In the previous studies, breadfruit leaf powder containing phenolics was reported to reduce antioxidant activity in layer [62] and broiler chickens [63]. The SOD enzyme prevents free hydroxyls by producing hydrogen peroxide and oxygen [64]. The low levels of endogenous SOD and MDA in quail meat suggest the potential of phenolics of breadfruit leaf powder as scavengers to reduce antioxidant activity. Moreover, the antioxidant activity decreased with improving quality of quail meat, emphasizing the findings of this study regarding the efficacy of breadfruit leaf powder.

## Conclusion

Dietary supplementation with breadfruit leaf powder was shown to improve growth performance, meat quality, and antioxidant activity in Japanese quail. Supplementation of the basal diet with 10 g/kg breadfruit leaf powder positively influenced meat color, drip loss, cooking loss, WHC, and meat tenderness. Although there were no significant differences in the levels of GPx, the improvements of SOD and MDA also indicated the efficacy of breadfruit leaf powder, perhaps due to its antioxidant compounds. These findings suggest the potential of breadfruit leaf powder as an alternative feed for improving the productivity and quality of quail meat.

## Authors’ Contributions

MTEP: Supervised the study. EPE, FF, and AP: Conducted the study. SK: Helped in the statistical analysis of the data. AP and SK: Helped in the visualization of tables and figures. AP and SC: Drafted the manuscript. MTEP and SC: Revised and submitted the manuscript. All authors read and approved the final manuscript.
